# Identification of bacteria in potential mutualism with toxic *Alexandrium catenella* in Chilean Patagonian fjords by *in vitro* and field monitoring

**DOI:** 10.1371/journal.pone.0301343

**Published:** 2024-06-04

**Authors:** Kyoko Yarimizu, Jorge I. Mardones, Javier Paredes-Mella, Ishara Uhanie Perera, So Fujiyoshi, Gonzalo Fuenzalida, Jacquelinne J. Acuña, Tay Ruiz-Gil, Marco Campos, Joaquin-Ignacio Rilling, Pedro Calabrano Miranda, Jonnathan Vilugrón, Oscar Espinoza-González, Leonardo Guzmán, Satoshi Nagai, Milko A. Jorquera, Fumito Maruyama

**Affiliations:** 1 Microbial Genomics and Ecology, The IDEC Institute, Hiroshima University, Higashi-Hiroshima City, Hiroshima, Japan; 2 Centro de Estudios de Algas Nocivas (CREAN), Instituto de Fomento Pesquero (IFOP), Puerto Montt, Chile; 3 Centro de Investigación en Recursos Naturales y Sustentabilidad (CIRENYS), Universidad Bernardo O’Higgins, Santiago, Chile; 4 Laboratorio Ecología Microbiana Aplicada (EMALAB), Scientific and Biotechnological Bioresource Nucleus (BIOREN-UFRO), Universidad de La Frontera, Temuco, Chile; 5 Laboratorio de Investigación en Salud de Precisión, Departamento de Procesos Diagnósticos y Evaluación, Facultad de Ciencias de la Salud, Universidad Católica de Temuco, Temuco, Chile; 6 Japan Coastal and Inland Fisheries Ecosystems Division, Fisheries Technology Institute, Japan Fisheries Research and Education Agency, Yokohama, Kanagawa, Japan; Macquarie University, AUSTRALIA

## Abstract

The dinoflagellate *Alexandrium catenella* is a well-known paralytic shellfish toxin producer that forms harmful algal blooms, repeatedly causing damage to Chilean coastal waters. The causes and behavior of algal blooms are complex and vary across different regions. As bacterial interactions with algal species are increasingly recognized as a key factor driving algal blooms, the present study identifies several bacterial candidates potentially associated with Chilean *Alexandrium catenella*. This research narrowed down the selection of bacteria from the Chilean *A. catenella* culture using antibiotic treatment and 16S rRNA metabarcoding analysis. Subsequently, seawater from two Chilean coastal stations, Isla Julia and Isla San Pedro, was monitored for two years to detect *Alexandrium* species and the selected bacteria, utilizing 16S and 18S rRNA gene metabarcoding analyses. The results suggested a potential association between *Alexandrium* species and Spongiibacteraceae at both stations. The proposed candidate bacteria within the Spongiibacteraceae family, potentially engaging in mutualistic relationships with *Alexandrium* species, included the genus of *BD1-7 clade*, *Spongiibbacter*, and *Zhongshania*.

## 1. Introduction

*Alexandrium catenella* is a toxin-producing phytoplankton species that has increasingly damaged coastal marine environments worldwide [1–3]. Since the first detection of this species in 1972 in the Magallanes region of Chile, its range has expanded slowly to the north, reaching the Aysén region in 1992, the southern Chiloé Island in the Los Lagos region in 2002, the Pacific coast of Chiloé Island in 2009, the coast of the Los Rios region in 2016, and recently the northern Bio-Bio region [4–11]. *Alexandrium catenella* produces paralytic shellfish toxins. The toxic cells first accumulate in bivalve tissues, leading to illness and death in higher trophic levels of organisms, including humans, upon digestion. These events bring severe implications to the local marine ecosystems and subsequently lead to coastal closure and restrictions on shellfish and salmon harvesting, which are the pillars of the Chilean economy [7, 8, 12, 13]. For example, the *A. catenella* bloom in the Aysén region in 2002 resulted in the loss of 1,800 metric tons of farmed salmon [14]. Similarly, a bloom in the Los Lagos region in 2006 impacted both the shellfish and salmon industries, causing losses equivalent to $9.2 million USD [7, 14, 15]. Two massive blooms of *A. catenella* and a dictyochophyceae of *Pseudochattonella verruculosa* in 2016 during the ‘El Niño Godzilla event’ affected 200 shellfish farms and 600 km of benthic artisanal fisheries and costed US$800 million in losses for the salmon industry [16, 17]. These impacts on the Chilean coastal waters by *A. catenella* blooms have increased over the past decade [10, 18].

The causes of harmful algal bloom (HABs) have been investigated mainly from the oceanographic factors such as water temperature, salinity, dissolved oxygen, phosphate, nitrogen, and silicate, namely physicochemical association as HAB factors. However, the potential role of bacteria in HAB formation, growth, and decline is also becoming a trending topic. Since Bell and Mitchell [19] reported in 1972 that bacterial communities inhabited around microalgal communities [20], studies have increasingly reported that algal–bacterial associations are particular and involve a complex exchange of nutrients and signaling molecules in their synergetic or antagonistic relationships [21–24]. In Chile, HAB research from an algal-bacterial mutualism perspective has just begun, and very few prior studies are available for bacterial interactions with Chilean *A. catenella*. For instance, in 2002, Córdova et al. [25] reported that the tentatively identified bacteria from a Chilean A. catenella exhibited strain-specific commensalism with several organisms, including *Aeromonas salmonicida*, *Flavobacterium breve*, *Pseudomonas diminuta*, *Pasteurella haemolytica*, *Proteus vulgaris*, *Pseudomonas putida*, *Pseudomonas versicularis*, and *Moraxella sp*. Uribe and Espejo [26] demonstrated that saprophytic bacteria enhanced the Chilean *A. catenella*’s toxicity five times more than the axenic culture. Amaro et al. [27] reported that three bacteria in a Chilean *A. catenella* culture released algal-lytic compounds, aminopeptidase, lipase, glucosaminidase, and alkaline phosphatase. Nevertheless, precise mechanisms of how specific bacteria interact with *A. catenella* in the HAB dynamics are still under investigation.

The aim of this study was to nominate potential bacteria associated with Chilean *A. catenella* from culture-based experiments and field monitoring and to provide information to help *A. catenella* bloom prediction and countermeasures from the bacteria point of view. We treated a Chilen strain of *A. catenella* culture with antibiotics to narrow the selection of bacteria possibly related to the strain and identified the taxonomy with 16S *rRNA* metabarcoding analysis. Specifically, the bacteria attached to *A. catenella* cells or cell walls likely migrate into the culture solution and are essential for algal growth [28–32]. Consequently, these bacteria were identified through the analysis. We then monitored the selected bacteria at two coastal stations, Isla San Pedro and Isla Julia, in the Chilean Patagonian fjords for two years.

## 2. Materials and methods

The materials and reagents used in this study are listed in Table 1.

**Table 1:** List of materials, reagents, and instrument.

Manufacture	Item	Part number
Sigma-Aldrich	antibiotics (penicillin/streptomycin/neomycin)	P4083-100 mL
	peptone	P-1265
MilliporeSigma	Sterivex GP 0.22μm filter unit	SVGP01050
	Whatman 1.0 μm pore-sized membrane	WHA111110
Fisher Scientific	trace metal-free hydrochloric acid	A466-250
	agar	BP1423-500
	Nunc™ Cell Culture Treated Flasks with Filter Caps	12-565-57
	sterile 8-strip 0.2 mL PCR tubes and flat cap	AB0451 & 4323032
	96-well 0.2 mL PCR plate and microseal film	4311971
	0.5 mL single tube	Q32856
	sterilized 1.5-mL tubes	2150N
	MiniAMP Plus Thermal Cycler	A37835
	Gel electrophoresis system	D2
	Qubit4TM Fluorometer	Q33238
	QubitTM dsDNA HS Assay kit	Q32851
	magnetic stand for 96 wells	AM10027
	TBE buffer	B52
	Pellet Pestle™ Cordless Motor	K749540-0000
Illumina	PhiX control Kit v3	FC-110-3001
	MiSeq Reagent Kit v3	MS-102-3003
	Nextera XT v2 Index Kit set A, B, C and D	FC-131-2001, 2002, 2003 and 2004
Promega	Blue/Orange Loading Dye 6X	G1881
	ProNex® Size-Selective Purification System	NG2002
Agilent Technologies	D1000 ScreenTape	5067–5582
	DNA markers D1000 Reagent	5067–5602
	loading tips specific for Agilent TapeStation systems	5067–5598
	Agilent Tapestation 4150	G2992AA
Takara Bio USA	Terra PCR Direct Polymerase Mix	639271
Integrated DNA Technologies	nuclease free water	11-05-01-04
Cleaver Scientific	agarose	CSL-AG500
MaestroGen	DNA Ladder	02001–500
Biotium	GelRed® Nucleic Acid Gel Stain	41002
Analytik Jena, AG	GelDoc-ItTS2 Imager	
Merck KGaA	ethanol	E7023
Roche	DNA polymerase KAPA HiFi Hotstart ReadyMix	KK2602
Merck KGaA)	Molecular grade NaOH	1091371000
Biobase Co.	Biobase PCR-800 PCR Cabinet	
Bio-Rad	Chelex® 100 Chelating Resin	1432832
Eppendorf	Centrifuge with A-8-17 rotor	5702

### 2.1 Growth media

All glass containers were soaked in 3 N hydrochloric acid for at least 2 days, rinsed with Milli-Q water, dried in a laminar-flow air bench, and autoclaved at 121°C for 30 minutes. Alternatively, sterile containers (Nunc cell culture-treated flasks with filter caps) were used. Seawater (SW) from Metri (-41.597; -72.7056, Los Lagos, Chile) was filtered through a 0.22-μm pore-sized membrane, mixed with 0.005% hydrochloric acid, and autoclaved at 121°C for 30 minutes. The average salinity of SW was confirmed to be 33, and the pH of the autoclaved SW ranged between 8.0 and 8.2 at ambient temperature. Sterile L1 nutrient [33] was added to the sterile SW to make a growth media per the manufacturer’s instructions.

### 2.2 Algal maintenance

*Alexandrium catenella* strain *CREAN AC11* was isolated from a cyst collected from Isla San Pedro in 2014 and used for the experiments herein. Cultures were maintained in SW+L1 in T25 cell culture flasks under 50 μmol photons m^−2^ s^−1^ on a 16:8 h light:dark cycle at 15±2°C (standard growth condition). The upper portion of a culture containing healthy cells was diluted to <500 cells mL^−1^ with fresh SW+L1 media every 3 weeks.

### 2.3 Bacteria screening from the *A. catenella* culture

All procedures were done in a laminar-flow hood with sterile devices. Fifty mL of *A. catenella* culture was filtered through a 0.22-μm membrane as a pre-antibiotic-treated sample (pre-treated sample), and the membrane was stored at −20°C for metabarcoding analysis.

**Fig 1:**
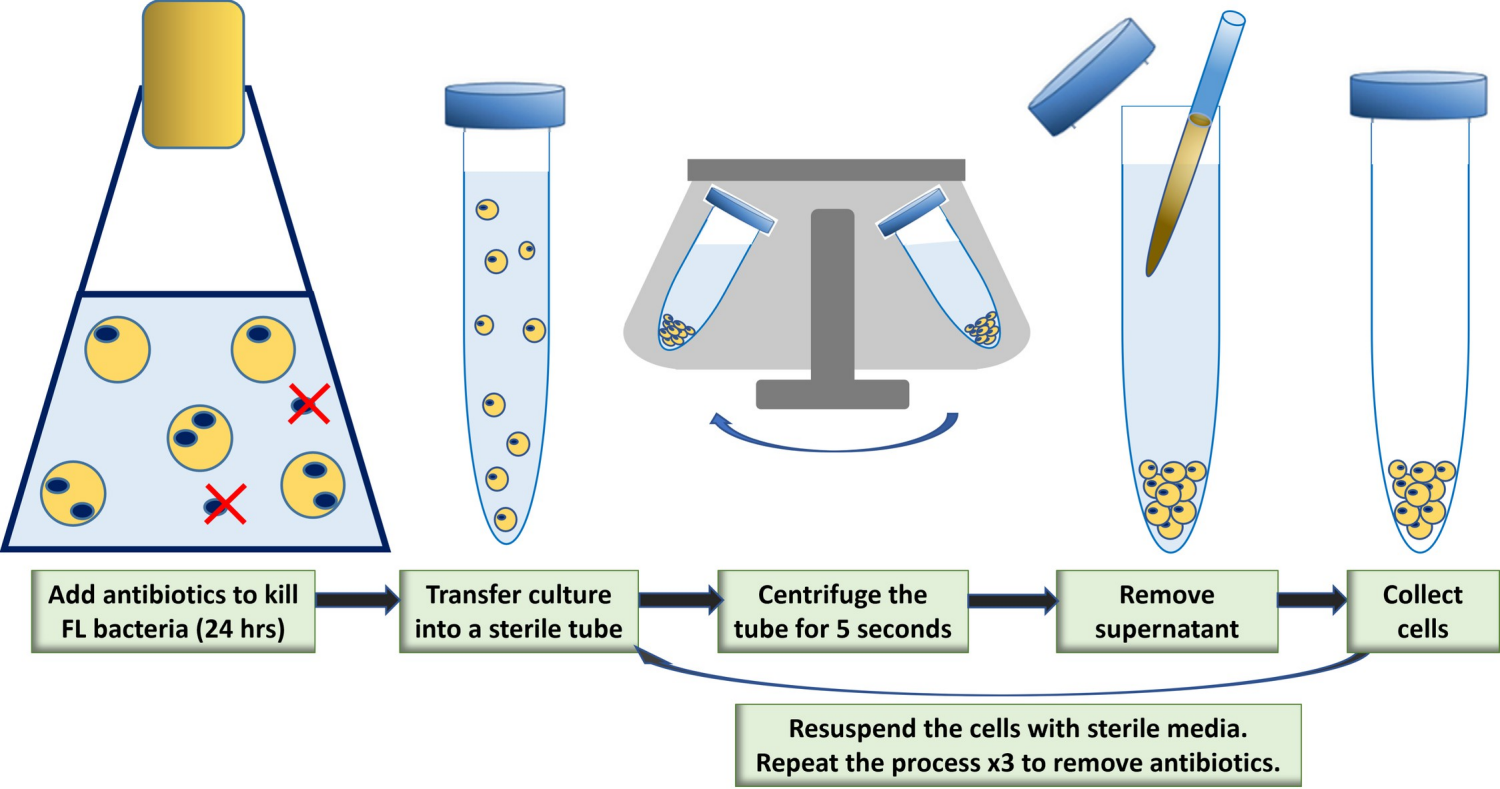
Schematic procedure of antibiotic treatment on *A. catenella*: Approximately 10^5^ cells L^−1^ of *A. catenella* in SW+L1 were treated with 0.1% (v/v) antibiotics for 24 hours under the standard growth condition. The culture was transferred into a 15-mL sterile tube and centrifuged at 2,290 G-force for five seconds to collect cell pellets. After removing the supernatant, the pellet was washed three times and re-suspended with SW+L1: Blue dot = free-living (FL) bacteria; Yellow circle = an *A. catenella* cell; Yellow circle with blue dots = particle-associated (PA) bacteria in an *A. catenella* cell.

A schematic procedure of antibiotic treatment on *A. catenella* is shown in Fig 1. Approximately 10^5^ cells L^−1^ of *A. catenella* in SW+L1 were treated with 0.1% (v/v) antibiotics (penicillin 5 units mL^−1^, streptomycin 5 μg mL^−1^, and neomycin 10 μg mL^−1^) for 24 hours under the standard growth condition. The culture was transferred into a 15- mL sterile tube and centrifuged at 2,290 G-force for five seconds to collect cell pellets. After removing the supernatant, the pellet was washed three times and re-suspended with SW+L1. The antibiotic-treated culture was tested for free of culturable bacterial by spreading 10-μL of the culture on a marine broth agar plate (5 g L^−1^ peptone, 1 g L^−1^ yeast extract, 15 g L^−1^ agar in 75% SW) followed by incubation at 25°C for 2–3 days. 1 L was immediately.

**Fig 2:**
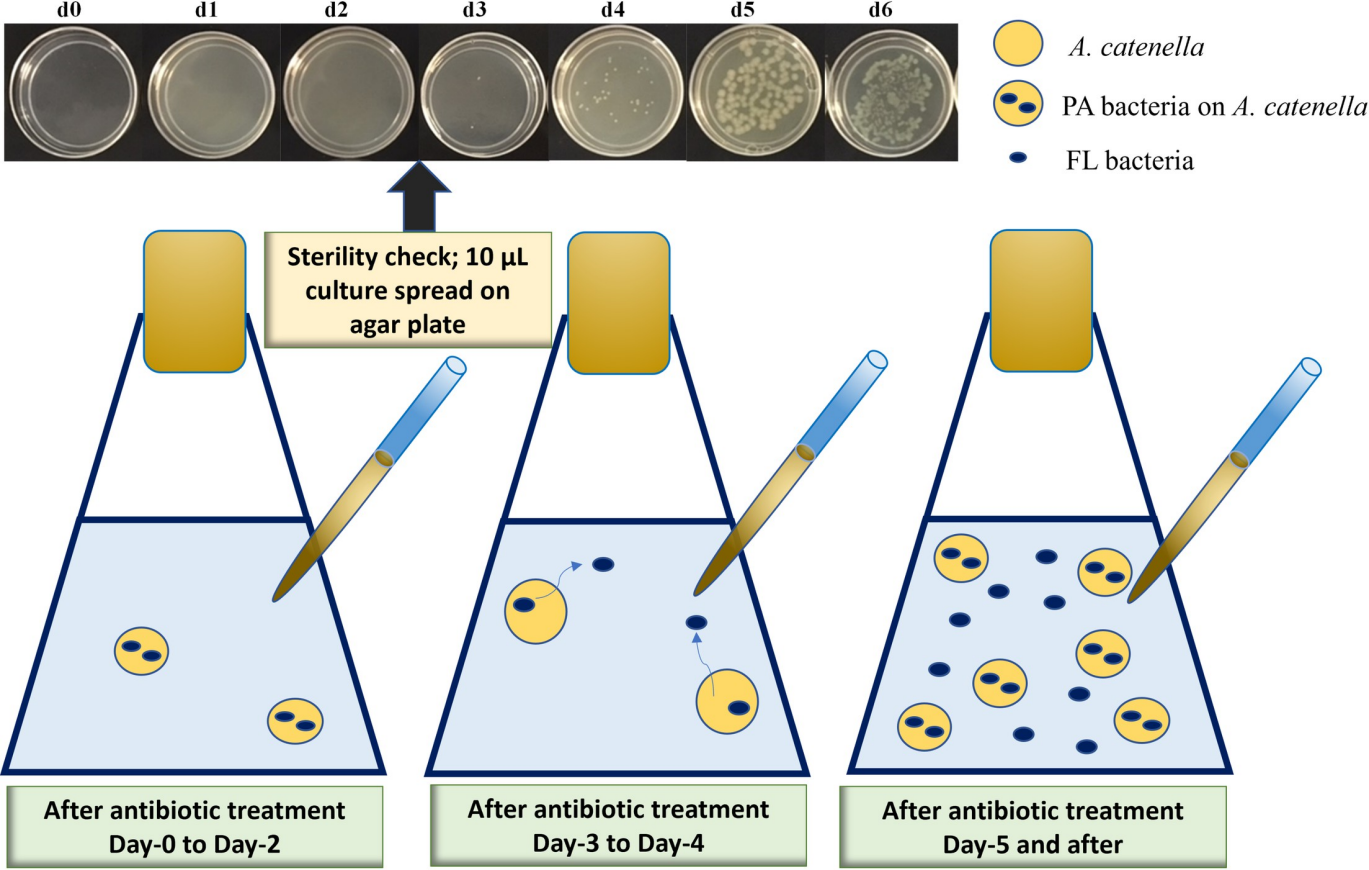
A schematic idea of bacterial growth in the antibiotic-treated *A. catenella* culture: A post-treated *A. catenella* culture was placed under the standard growth conditions. The culture was checked daily by gar plates. The culturable bacteria were observed in the 3-day-old algal solution, while a control solution (sterile media) remained bacteria-free.

The control for the agar plate test was the media without *A. catenella* cells. The agar plate test was performed daily on the post-antibiotic-treated culture, and it should be noted that bacteria reappeared in the *A. catenella* culture after three days (Fig 2). The culture was left to grow with the reappeared bacteria for 3 weeks. The upper portion of a culture containing healthy cells was diluted to <500 cells mL^−1^ with SW+L1 in a new sterile container and treated again with 0.1% (v/v) antibiotics for 24 hours. The cells were washed three times to remove antibiotics, re-suspended with sterile SW+L1 in a new sterile container, and then left to grow for 3 weeks. This process was repeated for five consecutive subcultures. The fifth subculture was grown for 3 weeks, and the total volume of the fifth culture (50 mL) was filtered through a 0.22-μm pore-sized membrane, which was stored at −20°C for metabarcoding analysis (post-treated sample).

### 2.4 Metabarcoding analysis

The membranes of the pre-treated and post-treated samples were processed for DNA extraction using the Chelex-buffer method [34]. The extracted DNA was processed for high-throughput amplicon sequencing with the primer set (Table 2) as detailed in the visual protocols [35, 36]. The obtained sequences were analyzed with DADA2 v.1.14.1 [38, 39]. Taxonomic identification was done on the sequences assigned to amplicon sequence variants (ASVs) against SSU Ref tree of SILVA release 132 and SILVA release 138 for 18S rRNA and 16S rRNA gene amplicon sequences, respectively [40]. For 16S rRNA sequences, singletons, mitochondria, and chloroplasts were removed (S1-S3 Tables in S1 File). The data were eliminated from the analysis when the total sequence reads were considerably low (Goods coverage below 99%), which could lead to bias in algal–bacterial correlation analysis.

**Table 2:** First PCR primers for metagenomic library preparation: The primers used for 16S rRNA sequencing are 16S-341F and 16S805R. The primers used for 18S rRNA sequencing are SSU-F1289 and SSU-R1772.

Primer Name	Target variable region	Overhang Adapter (5’→3’)	Region of Interest Specific Sequence (5’→ 3’)	Reference
16S-341F	16S rRNA V3–V4	ACA CTC TTT CCC TAC ACG ACG CTC TTC CGA TCT	CCT ACG GGN GGC WGC AG	[41]
16S-805R		GTG ACT GGA GTT CAG ACG TGT GCT CTT CCG ATC T	GAC TAC HVG GGT ATC TAA TCC	[41]
SSU-F1289	18S rRNA V7–9	ACA CTC TTT CCC TAC ACG ACG CTC TTC CGA TCT	TGG AGY GAT HTG TCT GGT TDA TTC CG	[42]
SSU-R1772		GTG ACT GGA GTT CAG ACG TGT GCT CTT CCG ATC T	TCA CCT ACG GAW ACC TTG TTA CG	[43]

### 2.5 Monitoring of algae and bacteria in the Isla San Pedro and Isla Julia

**Fig 3:**
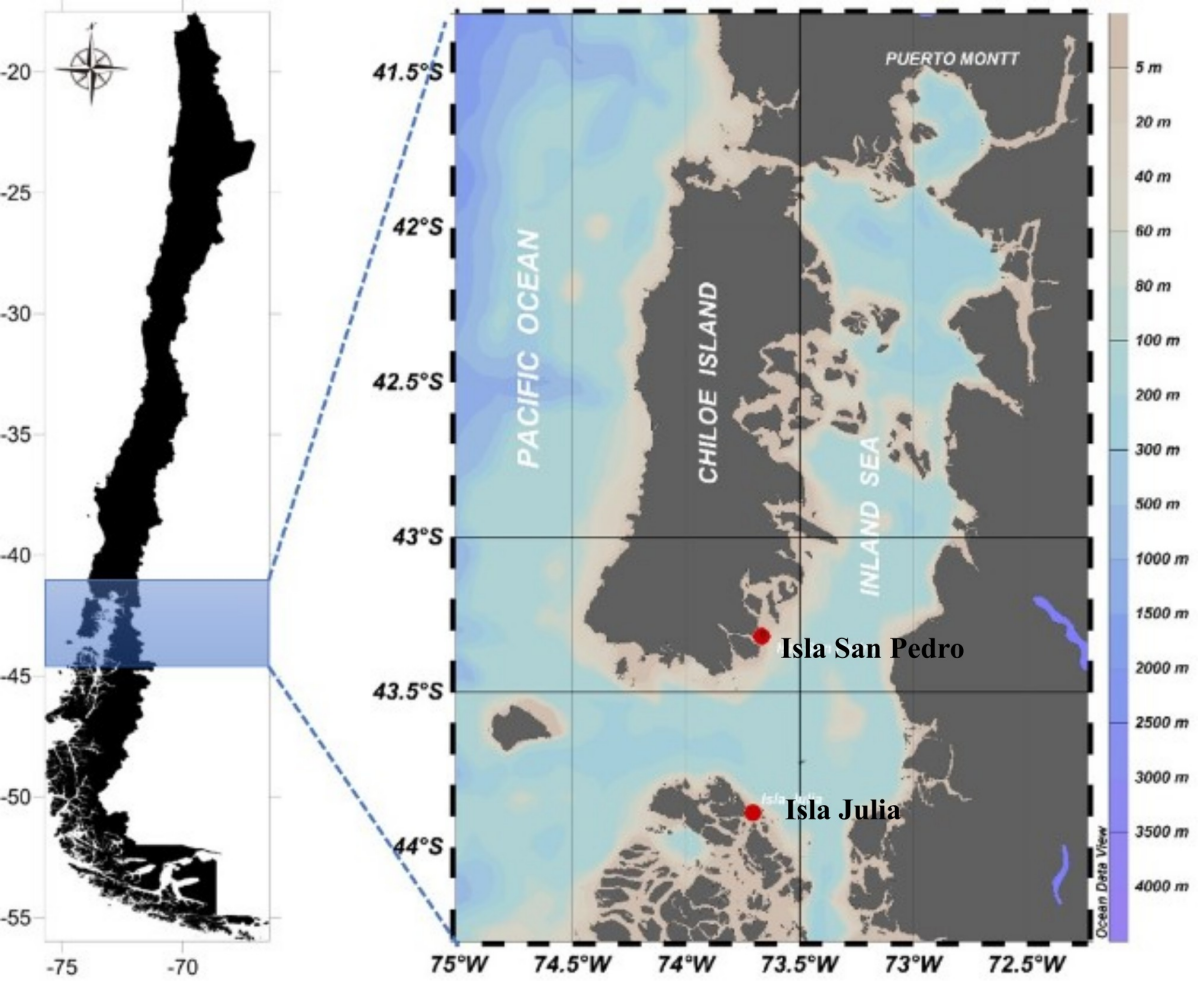
Map of sampling stations: The routine sampling was performed at the two stations, 1.5 km off the coast of Isla Julia (-43.901; -73.704, the lower red dot in the map) and 1 km off the coast of Isla San Pedro (-43.313; -73.662, the upper red dot in the map). This map was created with Ocean Data View (ODV) [37] retrieved in 2022 from https://odv.awi.de.

Water was collected at 1.5 km off the coast of Isla Julia (-43.901; -73.704) (hereafter “Isla Julia”) and 1 km off the coast of Isla San Pedro (-43.313; -73.662) (hereafter “Isla San Pedro”) biweekly from March 2019 to March 2021 (Fig 3). Sampling could not be performed between Apr 2020 and Jun 2020 owing to the lockdown mandate for the COVID-19 pandemic. Water was collected at a 10-m depth with a deployed conductivity–temperature–depth (CTD) sampler from a survey ship and transferred into a triple-washed plastic container. Samples were processed per the protocol of Yarimizu et al. [35]: 1 L was immediately filtered through a 1-μm filter membrane followed by a 0.22-μm membrane to separately collect larger bacteria/attached bacteria and smaller and freely present bacteria (hereafter particle-associated (PA) and free-living (FL) bacteria). To analyze the 18S rRNA gene, another 1 L of the sample was filtered through a 0.22-μm membrane. The membranes were stored in a freezer on the ship, and the metabarcoding analysis was performed in a laboratory (Section 2.4). For microscopic phytoplankton identification, 200 mL of the water sample was concentrated ×20 using a filter set. The concentrated samples (10 mL) were fixed with Lugol’s iodine to a final concentration of 1%. The Lugol-fixed samples were stored in a refrigerator until microscopic analysis was performed to identify and quantify algal genus names. Chlorophyll *a* (chl *a*) was measured by CTD during the sampling.

### 2.6 Statistical analysis

Pearson’s correlation was also used to determine the linear correlation between *Alexandrium* and bacteria. Furthermore, cross-correlations were used to elucidate any time-lagged relationships between *Alexandrium* and bacteria species that had a significant correlation by Pearson’s correlation. The statistical analyses were performed using R version 4.1.2 (R Core Team, 2021).

## 3. Results

### 3.1 Culture study

The *A. catenella* culture treated with antibiotics was initially free of culturable bacteria, but some culturable bacteria appeared in the algal solution within three days, while a control solution (sterile media) remained free of culturable bacteria (Fig 2). The bacteria in the pre-treated and post-treated cultures were compared by metabarcoding analysis (Table 3 and S1 Fig in S1 File). Proteobacteria displayed the dominant phyla in both pre- and post-treated cultures. Of the Proteobacteria, Gammaproteobacteria was the most abundant class, followed by Alphaproteobacteria and Deltaproteobacteria in both pre- and post-treated cultures. The relative abundance of Bacteroidetes and Deltaproteobacteria were increased in the post-treated culture. The notably increased (>5%) class of bacteria after treatment were Alteromonadaceae, Cyclobacteriaceae, Nannocystaceae, Spongiibacteraceae, and Thalassospiraceae.

**Table 3:** Bacteria composition in *A. catenella* culture before and after antibiotic treatment: The relative abundance of bacteria in the *A. catenella* culture was compared before and after antibiotic treatment by 16S rRNA gene metabarcoding assay.

Phylum	Pre, %	Post, %		Family	Pre, %	Post, %	
Actinobacteria	0.00	0.10	*	**Alteromonadaceae**	**18.80**	**49.90**	*
Bacteroidetes	24.53	13.87		Bacteriovoracaceae	0.43	0.01	
Planctomycetes	11.02	0.04		Balneolaceae	0.57	0.64	
Proteobacteria	64.39	85.91	*	Bdellovibrionaceae	1.35	0.00	
Verrucomicrobia	0.05	0.08	*	Chitinophagaceae	1.91	0.00	
				Coxiellaceae	0.00	0.73	
**Class**	**Pre, %**	**Post, %**		Cryomorphaceae	19.01	0.91	
Actinobacteria	0.00	0.10		**Cyclobacteriaceae**	**1.27**	**12.27**	*
Alphaproteobacteria	17.49	11.06		Flavobacteriaceae	1.77	0.04	
Bacteroidia	23.97	13.23		Kordiimonadaceae		0.56	
Deltaproteobacteria	1.78	11.16	*	Halieaceae	0.74	0.00	
Gammaproteobacteria	45.12	63.70	*	Hyphomonadaceae	9.84	0.00	
Phycisphaerae	9.39	0.00		Magnetospiraceae	0.44	2.97	*
Planctomycetacia	1.63	0.04		Marinobacteraceae	18.95	0.87	
Rhodothermia	0.57	0.64	*	Methyloligellaceae	0.37	0.00	
Verrucomicrobiae	0.05	0.08	*	Methylophagaceae	3.20	0.43	
				Methylophilaceae	0.05	0.00	
**Order**	**Pre, %**	**Post, %**		NA	2.64	0.43	
Alteromonadales	37.75	53.34	*	**Nannocystaceae**	**0.00**	**11.14**	*
Balneolales	0.57	0.68	*	Nisaeaceae	0.70	0.18	
Bdellovibrionales	1.78	0.01		Nocardiaceae	0.00	0.10	
Betaproteobacteriales	0.05	0.00	*	NS9_marine_group	0.00	0.01	
Caulobacterales	9.84	0.00		Oleiphilaceae	0.13	0.00	
Cellvibrionales	1.70	11.28	*	Opitutaceae	0.05	0.00	
Chitinophagales	1.91	0.00		Parvibaculaceae	0.18	0.20	
Corynebacteriales	0.00	0.10	*	Phycisphaeraceae	9.39	0.00	
Coxiellales	0.00	0.77	*	Pirellulaceae	0.05	0.04	
Cytophagales	1.27	12.89	*	Porticoccaceae	0.30	0.00	
Flavobacteriales	20.78	0.01		Pseudohongiellaceae	0.58	0.46	
KI89A_clade	0.25	0.00		Rhizobiaceae	0.33	0.00	
Kordiimonadaceae	0.00	0.59	*	Rhodobacteraceae	3.63	0.54	
Myxococcales	0.00	7.59	*	Rhodospirillaceae	0.09	0.00	
NA	1.29	0.02		Rubinisphaeraceae	0.23	0.00	
Nitrosococcales	3.20	0.45		Saccharospirillaceae	1.27	0.45	
NRL2	0.50	0.00		Solimonadaceae	0.19	0.09	
Oceanospirillales	1.98	0.96		Sphingomonadaceae	0.08	0.37	*
Opitutales	0.05	0.09	*	**Spongiibacteraceae**	**0.67**	**10.74**	*
Parvibaculales	0.27	0.09		**Thalassospiraceae**	**0.79**	**5.89**	*
Phycisphaerales	9.39	0.00		Vibrionaceae	0.00	0.01	*
Pirellulales	0.05	0.56	*				
Planctomycetales	0.29	0.00					
Puniceispirillales	0.20	0.00					
Rhizobiales	0.95	0.00					
Rhodobacterales	3.63	0.57					
Rhodospirillales	1.32	9.31	*				
Salinisphaerales	0.19	0.10					
Sphingomonadales	0.08	0.39	*				
Thalassobaculales	0.70	0.19					
Vibrionales	0.00	0.01	*				

(*) relative % increased after treatment.

### 3.2 Field study

#### 3.2.1 Detection of *Alexandrium spp*

**Fig 4:**
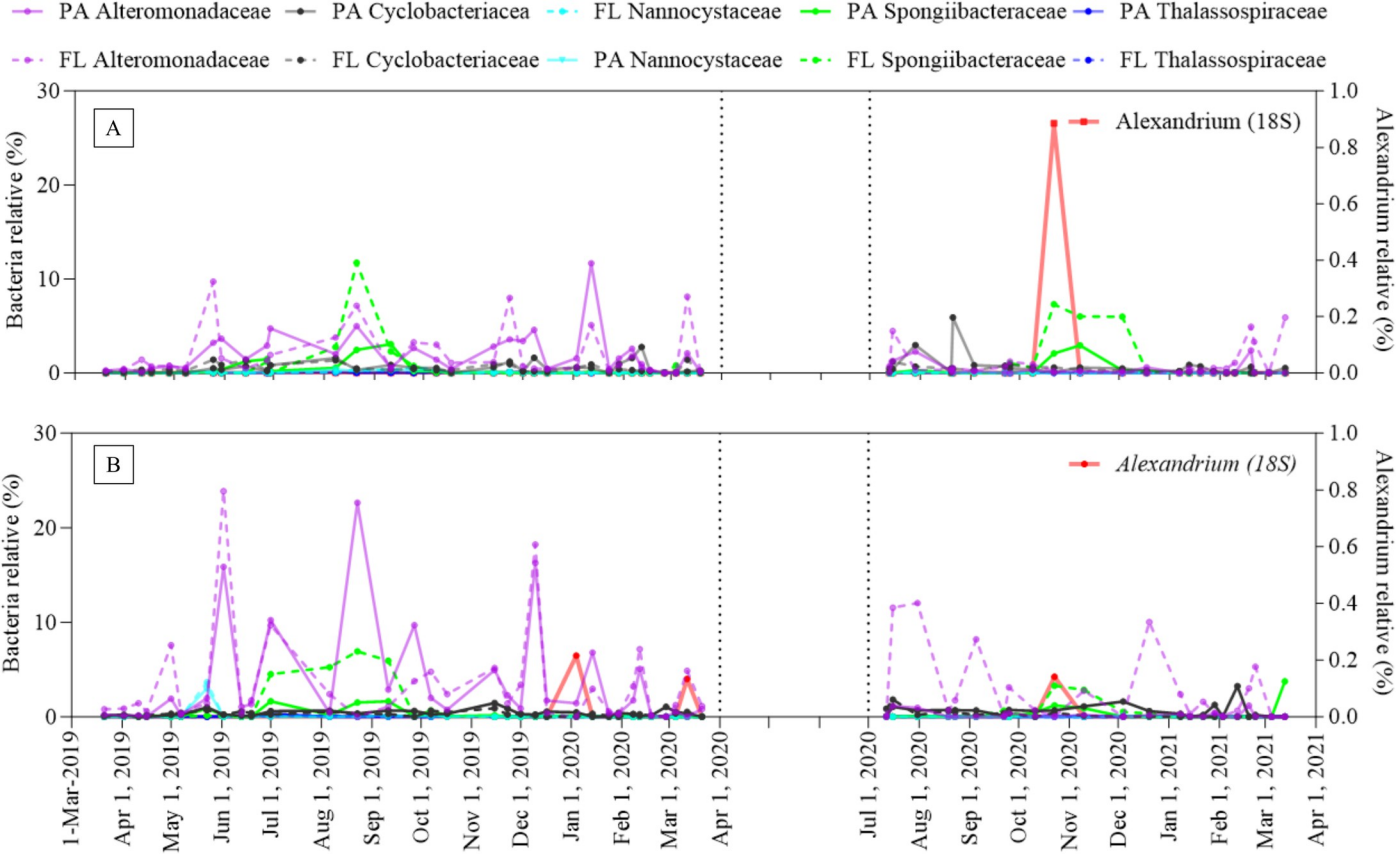
Relative abundance of target bacteria in Isla San Pedro and Isla Julia: The relative abundance of Alteromonadaceae, Cyclobacteriaceae, Nannocystaceae, Spongiibacteraceae, and Thalassospiraceae were monitored on the coast of A) Isla San Pedro and B) Isla Julia from March 2019 to March 2021 using 16S rRNA gene metabarcoding analysis (left Y-axis). Dinoflagellate *Alexandrium* species were monitored at the same stations using 18S rRNA gene metabarcoding analysis (right Y-axis).

*Alexandrium* spp. were monitored along the coasts of Isla San Pedro and Isla Julia for 2 years. No *Alexandrium* blooms occurred during this period. However, 18S rRNA gene metabarcoding analysis identified *Alexandrium* spp. in the Isla Julia water on 22 Oct 2020, comprising 0.9% of the total reads (Fig 4). Similarly, the metabarcoding analysis detected Alexandrium spp. in the Isla San Pedro water on the same day and at two additional time points (04 Jan 2020 and 12 Mar 2020). Yet, these relative abundances were approximately 0.1%, which is tenfold lower than that observed in Isla Julia (Fig 4). Microscopy at both stations could not detect *Alexandrium* cells, likely due to their very low absolute cell counts (S2 Fig in S1 File).

**Fig 5:**
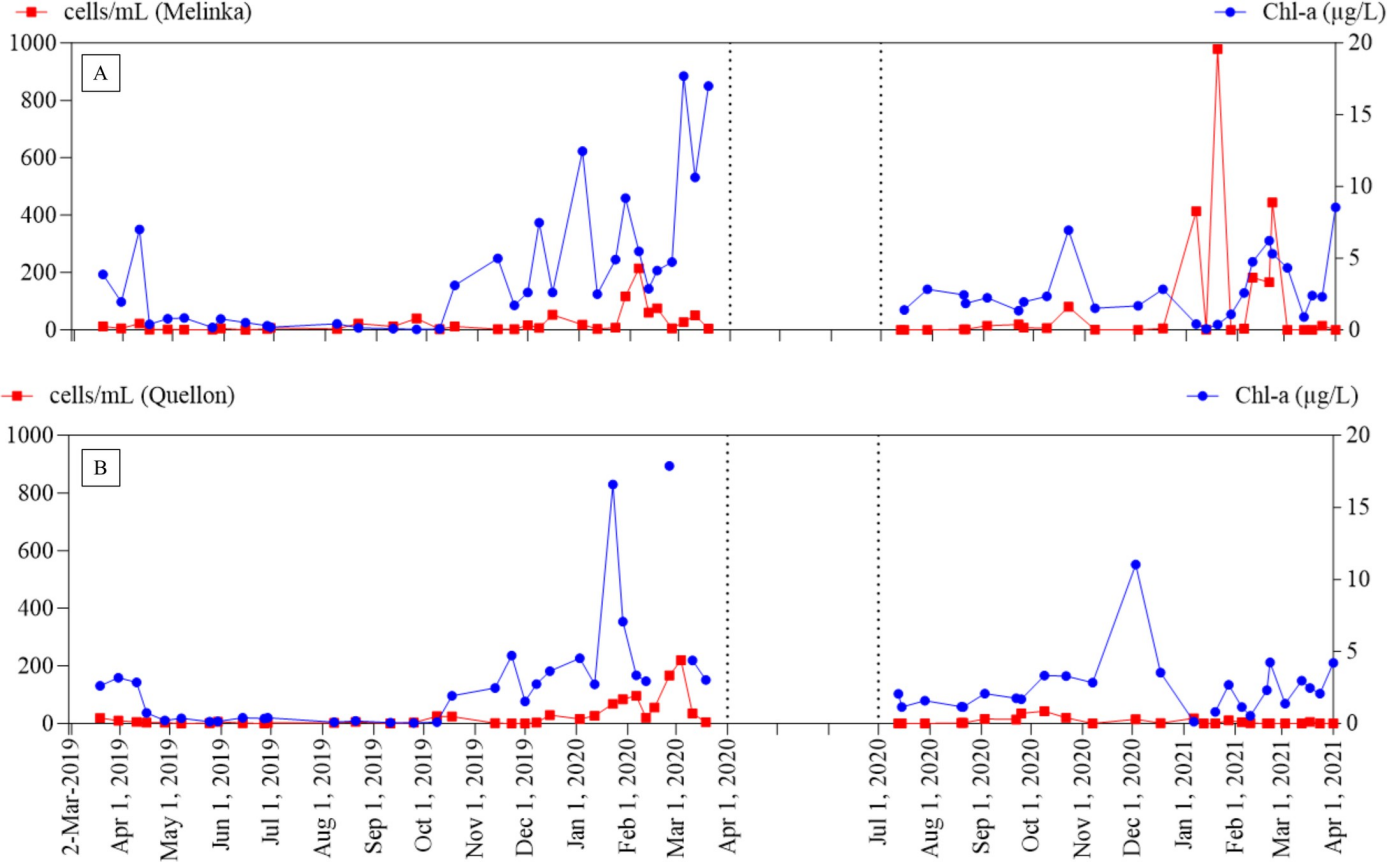
Monitoring of total phytoplankton cell counts and chl *a* in Isla San Pedro and Isla Julia: The phytoplankton species were identified by microscopy and cell counts were recorded. The figure shows total cell counts per mL of water sample in A) Isla Julia and B) Isla San Pedro from March 2019 to March 2021. Chl *a* was measured during each sampling using a CTD sampler.

To understand the algal biomass at the two stations, the total algal cell numbers was counted using microscopy and chl *a* with a CTD sampler. Both values generally increased during the austral summer, spanning from December to March (Fig 5). Thus, the detection of *Alexandrium* spp. in October at both stations using 18S rRNA metabarcoding (Fig 4) sindicates that *Alexandrium* spp. can be present in Isla Julia and Isla San Pedro outside of the prime season when phytoplankton actively proliferate.

#### 3.2.2 Detection of target bacteria

The classes of bacteria that notably increased in the culture after antibiotic treatment—Alteromonadaceae, Cyclobacteriaceae, Nannocystaceae, Spongiibacteraceae, and Thalassospiraceae—were monitored at Isla San Pedro and Isla Julia using 16S rRNA gene metabarcoding analysis over a span of 2 years. Among these five bacterial classes, Alteromonadaceae and Cyclobacteriaceae were sporadically detected throughout the study period, while Nannocystaceae and Thalassospiraceae were rarely observed (Fig 4). The relative abundance of Spongiibacteraceae increased simultaneously with that of *Alexandrium* spp. at both stations on 22 Oct 2020 (Fig 4). Pearson’s correlation analysis was conducted to evaluate the relationship between *Alexandrium* spp. and these bacterial classes, revealing a significant correlation between Spongiibacteraceae (FL and PA) and *Alexandrium* spp., particularly at Isla Julia (Table 4). Subsequently, cross-correlation analysis was applied to the Isla Julia dataset to ascertain any time lag between *Alexandrium* spp. and Spongiibacteraceae. No time-lagged relationship between Spongiibacteraceae and *Alexandrium* spp. was identified, suggesting that they increased and decreased simultaneously (S3 Fig in S1 File).

**Table 4:** Correlation between *A. catenella* and class of bacteria selected by culture screening: The class of bacteria selected by the culture study, Alteromonadaceae, Cyclobacteriaceae, Nannocystaceae, Spongiibacteraceae, and Thalassospiraceae were monitored at Isla Julia and Isla San Pedro for 2 years using 16S rRNA metabarcoding analysis. *Alexandrium* spp. was also monitored simultaneously using 18S rRNA metabarcoding analysis. Pearson’s correlation was used to determine the linear correlation between the *Alexandrium* and these classes of bacteria obtained from the field monitoring.

Isla Julia (class)	Pearson r	P (two-tailed)	Significant (alpha = 0.05)	Number of XY Pairs
FL Alteromonadaceae	-0.0865	0.5262	No	56
FL Cyclobacteriaceae	0.0577	0.6725	No	56
FL Nannocystaceae	-0.0404	0.7695	No	55
FL Spongiibacteraceae	0.4197	0.0013	Yes *	56
FL Thalassospiraceae	-0.0374	0.7844	No	56
PA Alteromonadaceae	-0.0898	0.5103	No	56
PA Cyclobacteriacea	-0.0753	0.5812	No	56
PA Nannocystaceae	-0.0634	0.6428	No	56
PA Spongiibacteraceae	0.3375	0.0110	Yes *	56
PA Thalassospiraceae	-0.0290	0.8322	No	56
Isla San Pedro (Class)	Pearson r	P (two-tailed)	Significant (alpha = 0.05)	Number of XY Pairs
FL Alteromonadaceae	-0.0995	0.4739	No	55
FL Cyclobacteriaceae	-0.0112	0.9353	No	55
FL Nannocystaceae	-0.0342	0.8041	No	55
FL Spongiibacteraceae	0.0535	0.7011	No	55
FL Thalassospiraceae	-0.0792	0.5653	No	55
PA Alteromonadaceae	-0.0799	0.5618	No	55
PA Cyclobacteriacea	0.0040	0.9770	No	55
PA Nannocystaceae	-0.0443	0.7484	No	55
PA Spongiibacteraceae	0.0546	0.6924	No	55
PA Thalassospiraceae	-0.0395	0.7748	No	55

FL = free-living, PA = particle associated, (*) correlated

#### 3.2.3 Candidates of bacteria genus potentially related to *Alexandriu spp*

**Fig 6:**
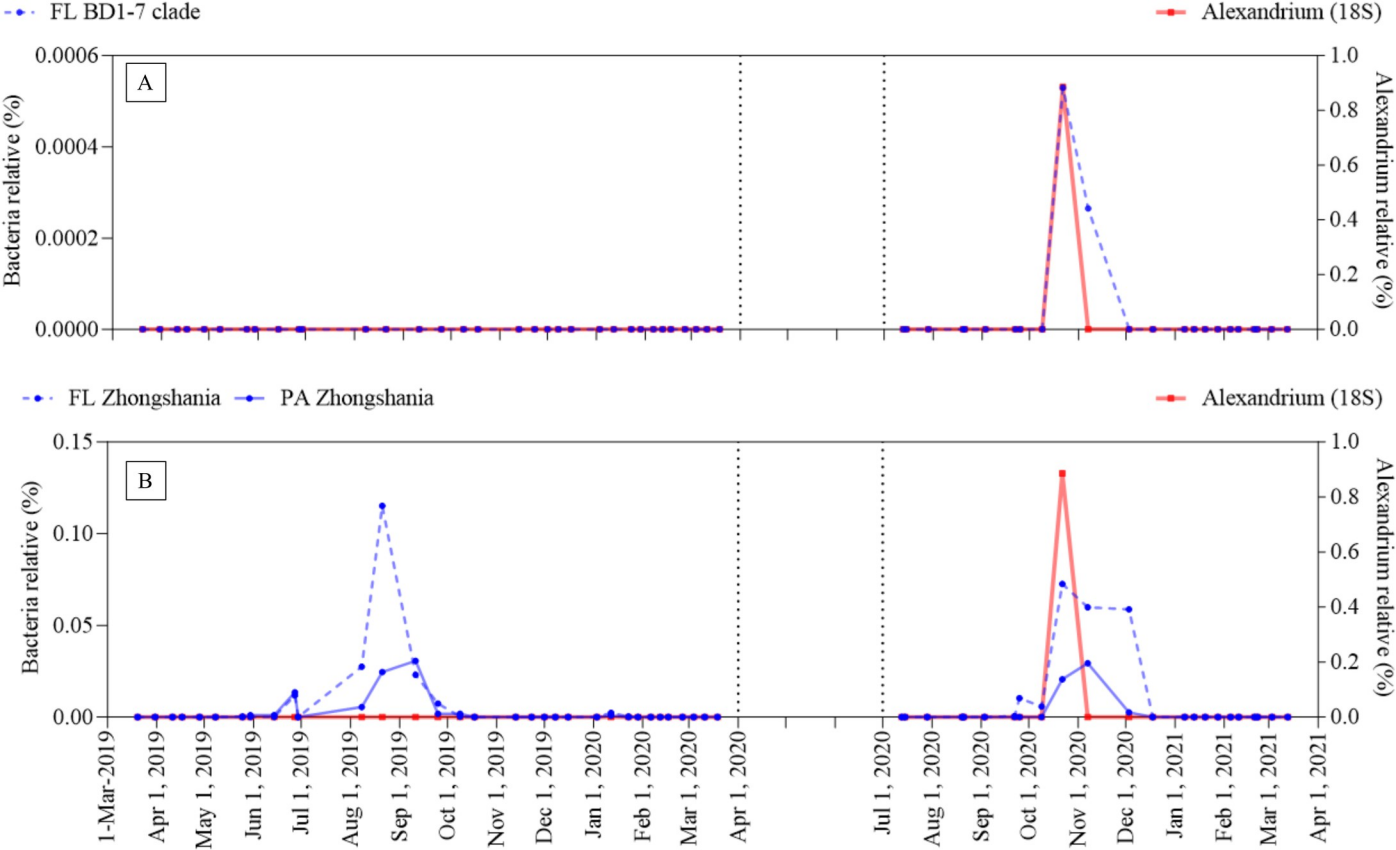
Time course relative abundance of *BD1-7 clade* and *Zhongshania* in Isla Julia: The relative abundance of BD1-7 clade and Zhongshania in Isla Julia were monitored from March 2019 to March 2021 using 16S rRNA gene metabarcoding analysis and plotted with that of *Alexandrium* spp.

Because there was a significant correlation between *Alexandrium* spp. and Spongiibacteraceae at Isla Julia (Table 4), Spongiibacteraceae in Isla Julia were further investigated at the genus level. Four genus taxonomies were assigned under Spongiibacteraceae in the Isla Julia dataset: *BD1-7 clade*, *Oceanicoccus*, *Spongiibacter*, and *Zhongshania*. Subsequently, Pearson’s correlation analysis was performed to determine the correlation between *Alexandrium* spp. and these subdivisions of Spongiibacteraceae (Table 5): A strong correlation was observed between *Alexandrium* spp. with the *BD1-7 clade* (FL) and *Zhongshania* (FL and PA). Although the relative abundance of *BD1-7* c*lade* (FL) was very low on 22 Oct 2020 when *Alexandrium* spp. was detected, its pattern of increase and decrease clearly aligned with that of *Alexandrium* spp. (Fig 6). *Zhongshania* (FL and PA) also coexisted with *Alexandrium* spp. on the same day, with relative of 0.08% and 0.02%, respectively (Fig 6). In Isla San Pedro, a strong correlation was also observed between *Alexandrium* spp. and *BD1-7 clade* (PA). While the genus *Spongiibacter* was detected at both stations, its correlation with *Alexandrium* spp. was not confirmed by Pearson’s correlation coefficient. Meanwhile, the Spongiibacteraceae that survived the culture experiment predominantly belonged to the genus *Spongiibacter*, as identified by the DADA2/SILVA statistical tools. Thus, an association between *Spongiibacter* and *A. catenella* remains a possibility (S4 Table in S1 File).

**Table 5:** Correlation between *A. catenella* and bacteria genera belonging to Spongiibcateracea in the field: The four bacteria genera belonging to Spongiibacteraceae were detected in Isla Julia and Isla San Pedro Pedro during the 2 years of monitoring using 16S rRNA metabarcoding analysis: *BD1-7 clade*, *Oceanicoccus*, *Spongiibacter*, *and Zhongshania*. Pearson’s correlation was used to determine the linear correlation between the *Alexandrium* and these bacteria genera.

Isla Julia (genus under Spongiibcateracea)	Pearson r	P (two-tailed)	Significant (alpha = 0.05)	Number of XY Pairs
FL BD1-7 clade	0.8927	<0.0001	**Yes**	55
FL Oceanicoccus	-0.0246	0.8584	No	55
FL Spongiibacter	-	-	-	55
FL Zhongshania	0.4227	0.0013	**Yes**	55
FL unknown	-0.0185	0.8932	No	55
PA BD1-7 clade	-0.0414	0.7618	No	56
PA Oceanicoccus	-	-	-	56
PA Spongiibacter	-0.0353	0.7959	No	56
PA Zhongshania	0.3514	0.0079	**Yes**	56
PA unknown	-0.0362	0.7910	No	56
Isla San Pedro (genus under Spongiibcateracea)	Pearson r	P (two-tailed)	Significant (alpha = 0.05)	Number of XY Pairs
FL BD1-7 clade	-0.0318	0.8175	No	55
FL Oceanicoccus	-0.0318	0.8175	No	55
FL Spongiibacter	-0.0318	0.8175	No	55
FL Zhongshania	-0.0079	0.9544	No	55
FL unknown	-0.0383	0.7814	No	55
PA BD1-7 clade	0.2757	0.0416	**Yes**	55
PA Oceanicoccus	-	-	-	
PA Spongiibacter	-0.0405	0.7691	No	55
PA Zhongshania	0.0520	0.7060	No	55
PA unknown	-0.0887	0.5194	No	55

FL = free-living, PA = particle associated. (-) is not applicable due to no assigned taxonomy.

## 4. Discussion

The interaction between algae and bacteria is highly complex, and understanding its mechanism cannot be achieved through short-term investigations. One primary reason is that their interaction is very specific and particular, varying from one strain to another. Moreover, even if an interaction is observed in a laboratory setting, it may not necessarily manifest the same way in natural environments. Natural environmental conditions are continually changing, posing significant challenges to verifying algal-bacterial interactions in the field. Therefore, it is crucial to uncover existing knowledge about these interactions, document previously unrecognized phenomena, and diligently accumulate insights for future reference. To this end, any discoveries related to algal-bacterial interactions hold value, and long-term field monitoring spanning several decades is essential to validate such findings.

The conventional understanding is that algal blooms are controlled by physical and chemical oceanic parameters, regardless of whether the sources are natural or anthropogenic. However, recent evidence has substantiated the relationship between bacteria and the algal bloom mechanism. Gajardo et al. [44] pointed out that understanding algal-bacterial interactions may offer a novel approach to predicting algal blooms, considering their significant impact on ecosystem biodiversity and productivity throughout their coevolutionary trajectories. Accordingly, this study focused on the selection of bacteria that may interact with the dinoflagellate of *A. catenella*, which has caused increasing damage along the Chilean coast. This study used the *A. catenella* culture prepared from the target site to screen bacteria by applying antibiotics and genomic analysis techniques. It is possible that these bacteria that survived the treatments simply favored the culture condition and selectively grew over the series of subcultures. However, our culture experiment showed that the *A. catenella* culture grew healthy with the surviving bacteria, which leaves the possibility that these bacteria may have a relationship with Chilean *A. catenella*. The class of bacteria, Alteromonadaceae, Cyclobacteriaceae, Nannocystaceae, Spongiibacteraceae, and Thalassospiraceae, remarkably increased after the culture screening process. Thus, these bacteria were monitored for two years at Isla Julia and Isla San Pedro in the Gulf of Corcovado in southern Chile, where salmon and shellfish harvesting are famous. In particular, the field monitoring at Isla Julia observed that the relative abundance of Spongiibacteraceae simultaneously increased and decreased with that of *Alexandrium* spp., suggesting that Spongiibacteraceae may have a certain interaction with this Chilean *A. catenella* strain.

At the time of this report, the family Spongiibacteraceae comprises six recognized genera: *Dasania*, *Marortus*, *Neomelitea*, *Sinobacterium*, *Spongiibacter*, and *Zhongshania*, as listed on [https://lpsn.dsmz.de/family/spongiibacteraceae]. Additionally, two other genera, *Oceanicoccus* and *clade BD1-7* have been reported. These bacterial genera have been isolated from marine waters, algae, and corals, yet their specific roles remain largely unknown [45]. Fu et al. noted that *Spongiibacter* was recently discovered in phycospheres associated with phytoplankton [46]. Yu et al. indicated that Spongiibacteraceae are commonly found in coral holobionts, playing a significant role in the global marine carbon cycle and energy metabolism [47]. Heins et al. identified a bacterial strain related to *clade BD1-7* in bloom samples and observed frequent occurrences of Spongiibacteraceae on particles [48]. Moreover, Spongiibacteraceae has been recognized as potential oil-degrading bacteria within microbial communities during oil spill experiments [49]. Further investigation of Spongiibacteraceae in relation to *A. catenella* could shed light on the mechanisms behind *A. catenella* blooms from an algal-bacterial mutualistic perspective. Subsequent research is warranted to explore the interactions between *A. catenella* and these bacteria, as well as the contribution of algal-bacterial interactions to *A. catenella* blooms.

One way to confirm the effect of bacterial species belonging to Spongiibacteraceae on *A. catenella* is to compare algal-bacterial co-cultures with algal axenic cultures. Our next objective is to prepare an axenic culture of this *A. catenella* strain and to perform binary culture experiments. We have yet to initiate the binary culture study. As widely recognized, a primary yet formidable challenge in binary culture studies lies in establishing axenic algal cultures. Completely removing bacteria adhering to algal cells proves exceedingly difficult, a challenge that numerous studies have highlighted concerning the preparation and maintenance of axenic algal cultures [28–32]. *Alexandrium catenella* is no exception to this difficulty [25, 41]. Furthermore, the potential for algae to be axenic varies by strain, complicating the process even further [32]. Maki and Imai [32] attempted to prepare axenic culture from five strains of *Heterocapsa circularisquama*, achieving success with only one strain. Some prior binary culture studies were conducted with so-called “axenic” cultures, noting the limitation of making a completely bacteria-free algal culture. However, to precisely understand the effects of bacteria on algae, having an axenic algal culture is fundamental. Thus, once we devise a method to render this *A. catenella* strain bacteria-free, our investigations will delve into algal-bacterial mutualisms. Specifically, we aim to identify signaling molecules and metabolites exchanged between *A. catenella* and specific Spongiibacteraceae species through metatranscriptome analyses. Furthermore, we hope to determine the mechanism behind how *A. catenella* and the bacteria benefit each other through the production and exchange of the molecules.

The difficulty of removing attached bacteria from algal cells implies that these attached bacteria might be essential for algal survival. This study observed that bacteria reappeared in the algal culture regardless of multiple antibiotic and washing processes, presumably migrating from the attached bacteria in the cell wall or inside the dinoflagellate [25, 26, 32]. Further investigation is required to understand the algal-bacterial relationship. In general, the roles of attached bacteria include stimulatory/inhibitory and synergetic/antagonistic effects. The stimulatory effect arises when bacteria provide nutrients to phytoplankton, particularly when the phytoplankton cannot photosynthesize due to limited light or nutrients. For example, bacteria were constantly observed in the cytoplasm and food vacuoles of *H*. *circularisquama* cells [32], and bacteria engulfed by food vacuoles of *Uroglena americana* reportedly provided essential phospholipids for the algal growth under nutrient-deficient conditions [50]. There are reports about bacterial contributions to vitamin B and iron uptakes of dinoflagellate *Lingulodinium polyedrum* [51, 52]. There is also a report about the synergetic interaction between *Pseudonitzchia multiseries* and *Sulfitobacter* by exchanging hormones, indole-3-acetic acid, ammonia, and organosulfur [22]. In contrast, the bacterial inhibitory role pertains to the decline and extinction of algal biomass resulting from the presence of algicidal bacteria and antagonistic interactions between bacteria and algae; such bacteria have also been reported [53, 54]. Mayali and Azam [55] stated that bacteria could swim to the surface of algal cells, use their hydrolases, and subsequently kill the metabolically coupled algal species. It is unclear how bacterial species of Spongiibacteraceae interact with the Chilean *A. catenella* strain at this point. However, we speculated that certain species of Spongiibacteraceae, potentially subdivisions of *BD1-7 clade* and *Zhongshania*, may exert a stimulatory or synergistic effect on *A. catenella*. This speculation arises because these bacteria were detected along with *A. catenella* in both Isla Julia and Isla San Pedro, and a positive correlation was demonstrated through statistical analysis. Similarly, there might be a stimulatory or synergistic effect on *A. catenella* from *Spongiibacter*, given their simultaneous presence at both stations and the persistence of a high relative abundance of *Spongiibacter* in the post-treated culture.

Several other noteworthy observations were obtained in this study. The dominant class of bacteria in the *A. catenella* was Gammaproteobacteria, which is often associated with many algal cultures, particularly the dinoflagellates [21, 22, 30, 56]. The most dominant bacterial genus in the post-treated *A. catenella* culture was the genus *Paraglaciecola* (S4 Table in S1 File). These are reportedly cold-adapted marine bacteria that have been isolated from marine algae, seagrasses, Arctic sea ice algae, and the diatom *Thalassiosira rotula* [57–60]. Given that Isla Julia and Isla San Pedro, where this *A. catenella* strain was isolated, experience relatively cold temperatures throughout the year (averaging 10–12°C), it remains plausible that *Paraglaciecola* and *A. catenella* share a mutualistic relationship. However, while this association was observed in the culture study, it wasn’t confirmed in the field study. Alternatively, *Paraglaciecola* might simply exhibit high resistance or remain inaccessible to the antibiotics, making them appear more abundant in culture than they truly are in their natural state. Continued monitoring of *Paraglaciecola* as a potential mutualistic bacterium for *A. catenella* is essential for confirmation.

Our ultimate goal is to integrate bacterial information into existing bloom prediction models, allowing for the prediction of *A. catenella* blooms from a bacterial perspective. Over the two years of monitoring, there were no *A. catenella* blooms detected at Isla Julia and Isla San Pedro, nor were *A. catenella* cells identified through microscopy. The *Alexandrium* data presented in this study were identified using 18S rRNA metagenomic analysis. This method has proven invaluable, revealing an identification that microscopy was not able to detect. With that said, using such a sporadic *A. catenella* data, constructing a bloom prediction model remains challenging. Continued monitoring of Isla Julia and Isla San Pedro is essential to accumulate sufficient data for a robust *A. catenella* prediction model.

In the meantime, we plan to incorporate an orthogonal absolute detection method for species of *Spongiibacteraceae* and *A. catenella*, such as Real-time PCR, in addition to the current microscopic method. The metabarcoding analysis utilized in this study is a groundbreaking molecular technique that can identify hundreds of ASVs of bacteria and eukaryotes from a single seawater sample, even within a complex, low-abundance community that is not conducive to conventional microscopy [16]. However, its primary application remains centered on relative detection [61, 62]. Incorporating real-time PCR or digital PCR to quantify species of *Spongiibacteraceae* and *A. catenella* could offer deeper insights into algal-bacterial mutualism.

## 5. Conclusion

This study evidenced a potential association between *Alexandrium* species and *Spongiibacteraceae* in Isla Julia and Isla San Pedro in southern Chile, based on both culture-based laboratory studies and two years of field monitoring. The suggested candidate bacteria within Spongiibacteraceae, possibly mutualistic with *Alexandrium* species, include the genus of *BD1-7 clade*, *Spongiibacter*, and *Zhongshania*. Further investigations are needed to confirm this association. However, gaining more knowledge about these bacteria could provide new insights into *A. catenella* blooms from an algal–bacterial perspective.

## Supporting information

S1 File (DOCX)
