# Supplementary material for: Identification of bacteria in potential mutualism with toxic Alexandrium catenella in Chilean Patagonian fjords by in vitro and field monitoring
Source: PLoS One. 2024 Jun 4;19(6):e0301343. doi: 10.1371/journal.pone.0301343 (PMC11149857; doi:10.1371/journal.pone.0301343)

**Identification of bacteria in potential mutualism with toxic *Alexandrium catenella* in Chilean Patagonian fjords by in vitro and field monitoring**

**Supporting Information**

**S1 Table. Sequence summary – 18S rRNA metabarcoding analysis performed on Isla Julia (right) and Isla San Pedro (left) waters**

| **ID** | **input** | **final** | **samples** |  | **ID** | **input** | **final** | **samples** |
| --- | --- | --- | --- | --- | --- | --- | --- | --- |
| SP-18S-02-01 | 11769 | 3874 | 21-Mar-2019 |  | J-18S-02-01 | 107801 | 55233 | 20-Mar-2019 |
| SP-18S-02-02 | 135631 | 46721 | 2-Apr-2019 |  | J-18S-02-02 | 90015 | 45926 | 31-Mar-2019 |
| SP-18S-02-03 | 80655 | 21301 | 11-Apr-2019 |  | J-18S-02-03 | 100791 | 61791 | 11-Apr-2019 |
| SP-18S-02-04 | 67803 | 21817 | 16-Apr-2019 |  | J-18S-02-04 | 147220 | 76280 | 17-Apr-2019 |
| SP-18S-02-05 | 18869 | 6966 | 1-May-2019 |  | J-18S-02-05 | 122308 | 63892 | 28-Apr-2019 |
| SP-18S-02-06 | 83261 | 16232 | 7-May-2019 |  | J-18S-02-06 | 120130 | 75523 | 8-May-2019 |
| SP-18S-02-07 | 71107 | 18375 | 23-May-2019 |  | J-18S-02-07 | 78303 | 45322 | 25-May-2019 |
| SP-18S-02-08 | 75639 | 24302 | 2-Jun-2019 |  | J-18S-02-08 | 112516 | 61807 | 30-May-2019 |
| SP-18S-02-09 | 109634 | 49362 | 13-Jun-2019 |  | J-18S-02-09 | 107666 | 48061 | 14-Jun-2019 |
| SP-18S-02-10 | 113236 | 47757 | 19-Jun-2019 |  | J-18S-02-10 | 113644 | 49206 | 27-Jun-2019 |
| SP-18S-02-11 | 112095 | 31753 | 1-Jul-2019 |  | J-18S-02-11 | 133029 | 53460 | 29-Jun-2019 |
| SP-18S-02-12 | 119252 | 47762 | 6-Aug-2019 |  | J-18S-02-12 | 73199 | 36333 | 8-Aug-2019 |
| SP-18S-02-13 | 36486 | 17304 | 23-Aug-2019 |  | J-18S-02-13 | 100833 | 53445 | 21-Aug-2019 |
| SP-18S-02-14 | 47023 | 16510 | 11-Sep-2019 |  | J-18S-02-14 | 64457 | 23265 | 11-Sep-2019 |
| SP-18S-02-15 | 38001 | 9106 | 27-Sep-2019 |  | J-18S-02-15 | 54641 | 23708 | 25-Sep-2019 |
| SP-18S-02-16 | 63422 | 19744 | 7-Oct-2019 |  | J-18S-02-16 | 29406 | 7572 | 9-Oct-2019 |
| SP-18S-02-17 | 60321 | 33873 | 17-Oct-2019 |  | J-18S-02-17 | 59520 | 33221 | 18-Oct-2019 |
| SP-18S-02-18 | 49612 | 26801 | 15-Nov-2019 |  | J-18S-02-18 | 57471 | 35173 | 13-Nov-2019 |
| SP-18S-02-19 | 167 | 26 | 23-Nov-2019 |  | J-18S-02-19 | 48890 | 29349 | 23-Nov-2019 |
| SP-18S-02-20 | 44006 | 22768 | 1-Dec-2019 |  | J-18S-02-20 | 58126 | 28976 | 1-Dec-2019 |
| SP-18S-02-21 | 19437 | 9551 | 10-Dec-2019 |  | J-18S-02-21 | 68446 | 37436 | 8-Dec-2019 |
| SP-18S-02-22 | 17575 | 10382 | 17-Dec-2019 |  | J-18S-02-22 | 26667 | 15398 | 16-Dec-2019 |
| SP-18S-02-23 | 23096 | 12080 | 4-Jan-2020 |  | J-18S-02-23 | 40149 | 20816 | 3-Jan-2020 |
| SP-18S-02-24 | 31981 | 13943 | 14-Jan-2020 |  | J-18S-02-24 | 24240 | 13652 | 12-Jan-2020 |
| SP-18S-02-25 | 67416 | 35709 | 24-Jan-2020 |  | J-18S-02-25 | 25166 | 13483 | 23-Jan-2020 |
| SP-18S-02-26 | 68963 | 32925 | 30-Jan-2020 |  | J-18S-02-26 | 80305 | 44736 | 29-Jan-2020 |
| SP-18S-02-27 | 69463 | 41341 | 8-Feb-2020 |  | J-18S-02-27 | 71850 | 37943 | 6-Feb-2020 |
| SP-18S-02-28 | 71055 | 33281 | 12-Feb-2020 |  | J-18S-02-28 | 63564 | 30927 | 12-Feb-2020 |
| SP-18S-02-29 | 80542 | 43576 | 19-Feb-2020 |  | J-18S-02-29 | 118517 | 38888 | 17-Feb-2020 |
| SP-18S-02-30 | 70716 | 35987 | 28-Feb-2020 |  | J-18S-02-30 | 30708 | 19283 | 26-Feb-2020 |
| SP-18S-02-31 | 60975 | 28460 | 5-Mar-2020 |  | J-18S-02-31 | 38582 | 20674 | 4-Mar-2020 |
| SP-18S-02-32 | 35603 | 14957 | 12-Mar-2020 |  | J-18S-02-32 | 44114 | 25043 | 11-Mar-2020 |
| SP-18S-02-33 | 56980 | 25494 | 21-Mar-2020 |  | J-18S-02-33 | 63941 | 29089 | 19-Mar-2020 |
| SP-18S-02-34 | 41448 | 17635 | 12-Jul-2020 |  | J-18S-02-34 | 35952 | 17911 | 13-Jul-2020 |
| SP-18S-02-35 | 24920 | 16054 | 16-Jul-2020 |  | J-18S-02-35 | 28825 | 12892 | 15-Jul-2020 |
| SP-18S-02-36 | 41217 | 16508 | 31-Jul-2020 |  | J-18S-02-36 | 88690 | 42117 | 29-Jul-2020 |
| SP-18S-02-37 | 49386 | 21989 | 19-Aug-2020 |  | J-18S-02-37 | 88261 | 38929 | 20-Aug-2020 |
| SP-18S-02-38 | 80240 | 33897 | 23-Aug-2020 |  | J-18S-02-38 | 106901 | 52789 | 21-Aug-2020 |
| SP-18S-02-39 | 65098 | 26851 | 5-Sep-2020 |  | J-18S-02-39 | 55839 | 32640 | 3-Sep-2020 |
| SP-18S-02-40 | 56433 | 29228 | 22-Sep-2020 |  | J-18S-02-40 | 54194 | 28452 | 22-Sep-2020 |
| SP-18S-02-41 | 57043 | 31222 | 25-Sep-2020 |  | J-18S-02-41 | 39712 | 22785 | 25-Sep-2020 |
| SP-18S-02-42 | 21100 | 8671 | 11-Oct-2020 |  | J-18S-02-42 | 39119 | 12410 | 9-Oct-2020 |
| SP-18S-02-43 | 51359 | 28401 | 23-Oct-2020 |  | J-18S-02-43 | 57585 | 33219 | 22-Oct-2020 |
| SP-18S-02-44 | 55315 | 23132 | 10-Nov-2020 |  | J-18S-02-44 | 58703 | 29548 | 7-Nov-2020 |
| SP-18S-02-45 | 86149 | 44082 | 4-Dec-2020 |  | J-18S-02-45 | 54551 | 26750 | 3-Dec-2020 |
| SP-18S-02-46 | 35474 | 12651 | 20-Dec-2020 |  | J-18S-02-46 | 37319 | 17827 | 18-Dec-2020 |
| SP-18S-02-47 | 31309 | 6455 | 8-Jan-2021 |  | J-18S-02-47 | 45336 | 21179 | 7-Jan-2021 |
| SP-18S-02-48 | 26064 | 13160 | 14-Jan-2021 |  | J-18S-02-48 | 32585 | 17091 | 13-Jan-2021 |
| SP-18S-02-49 | 53928 | 11691 | 22-Jan-2021 |  | J-18S-02-49 | 31640 | 15372 | 20-Jan-2021 |
| SP-18S-02-50 | 73905 | 11890 | 29-Jan-2021 |  | J-18S-02-50 | 50388 | 16953 | 28-Jan-2021 |
| SP-18S-02-51 | 32452 | 12384 | 3-Feb-2021 |  | J-18S-02-51 | 183948 | 4268 | 5-Feb-2021 |
| SP-18S-02-52 | 25695 | 10932 | 12-Feb-2021 |  | J-18S-02-52 | 41108 | 16776 | 10-Feb-2021 |
| SP-18S-02-53 | 26489 | 12582 | 19-Feb-2021 |  | J-18S-02-53 | 35323 | 18639 | 20-Feb-2021 |
| SP-18S-02-54 | 34425 | 17608 | 23-Feb-2021 |  | J-18S-02-54 | 21573 | 10228 | 22-Feb-2021 |
| SP-18S-02-55 | 10070 | 0 | 3-Mar-2021 |  | J-18S-02-55 | 6740 | 104 | 3-Mar-2021 |
| SP-18S-02-56 | 5261 | 25 | 13-Mar-2021 |  | J-18S-02-56 | 6518 | 53 | 13-Mar-2021 |

**S2 Table. Sequence summary – 16S rRNA metabarcoding analysis (free-living bacteria) performed on Isla Julia (right) and Isla San Pedro (left) waters**.

| **ID** | **input** | **final** | **samples** |  | **ID** | **input** | **final** | **samples** |
| --- | --- | --- | --- | --- | --- | --- | --- | --- |
| SP-16S-02-01 | 111348 | 78397 | 21-Mar-2019 |  | J-16S-02-01 | 39015 | 28270 | 20-Mar-2019 |
| SP-16S-02-02 | 114953 | 77994 | 2-Apr-2019 |  | J-16S-02-02 | 59073 | 41604 | 31-Mar-2019 |
| SP-16S-02-03 | 132330 | 89255 | 11-Apr-2019 |  | J-16S-02-03 | 53315 | 36626 | 11-Apr-2019 |
| SP-16S-02-04 | 108070 | 72972 | 16-Apr-2019 |  | J-16S-02-04 | 68348 | 46743 | 17-Apr-2019 |
| SP-16S-02-05 | 92758 | 63530 | 1-May-2019 |  | J-16S-02-05 | 88499 | 57855 | 28-Apr-2019 |
| SP-16S-02-06 | 123504 | 78349 | 7-May-2019 |  | J-16S-02-06 | 92006 | 62796 | 8-May-2019 |
| SP-16S-02-07 | 97831 | 64708 | 23-May-2019 |  | J-16S-02-07 | 144993 | 99238 | 25-May-2019 |
| SP-16S-02-08 | 121918 | 85116 | 2-Jun-2019 |  | J-16S-02-08 | 91786 | 62204 | 30-May-2019 |
| SP-16S-02-09 | 66670 | 44258 | 13-Jun-2019 |  | J-16S-02-09 | 80703 | 54886 | 14-Jun-2019 |
| SP-16S-02-10 | 77497 | 51775 | 19-Jun-2019 |  | J-16S-02-10 | 56044 | 36704 | 27-Jun-2019 |
| SP-16S-02-11 | 122183 | 83251 | 1-Jul-2019 |  | J-16S-02-11 | 114622 | 78763 | 29-Jun-2019 |
| SP-16S-02-12 | 105424 | 69391 | 6-Aug-2019 |  | J-16S-02-12 | 58154 | 38731 | 8-Aug-2019 |
| SP-16S-02-13 | 110951 | 41976 | 23-Aug-2019 |  | J-16S-02-13 | 57561 | 24940 | 21-Aug-2019 |
| SP-16S-02-14 | 131812 | 60197 | 11-Sep-2019 |  | J-16S-02-14 | 106522 | 50509 | 11-Sep-2019 |
| SP-16S-02-15 | 4879 | 1830 | 27-Sep-2019 |  | J-16S-02-15 | 64023 | 29483 | 25-Sep-2019 |
| SP-16S-02-16 | 36899 | 16061 | 7-Oct-2019 |  | J-16S-02-16 | 38717 | 18385 | 9-Oct-2019 |
| SP-16S-02-17 | 58427 | 39926 | 17-Oct-2019 |  | J-16S-02-17 | 81454 | 60371 | 18-Oct-2019 |
| SP-16S-02-18 | 12135 | 7052 | 15-Nov-2019 |  | J-16S-02-18 | 128700 | 95421 | 13-Nov-2019 |
| SP-16S-02-19 | 53138 | 38926 | 23-Nov-2019 |  | J-16S-02-19 | 73371 | 54320 | 23-Nov-2019 |
| SP-16S-02-20 | 80067 | 55702 | 1-Dec-2019 |  | J-16S-02-20 | 130829 | 95210 | 1-Dec-2019 |
| SP-16S-02-21 | 82551 | 55760 | 10-Dec-2019 |  | J-16S-02-21 | 82617 | 62110 | 8-Dec-2019 |
| SP-16S-02-22 | 51167 | 37852 | 17-Dec-2019 |  | J-16S-02-22 | 128912 | 93460 | 16-Dec-2019 |
| SP-16S-02-23 | 86 | 15 | 4-Jan-2020 |  | J-16S-02-23 | 69298 | 37753 | 3-Jan-2020 |
| SP-16S-02-24 | 10985 | 7783 | 14-Jan-2020 |  | J-16S-02-24 | 85551 | 63428 | 12-Jan-2020 |
| SP-16S-02-25 | 190683 | 131472 | 24-Jan-2020 |  | J-16S-02-25 | 40997 | 29301 | 23-Jan-2020 |
| SP-16S-02-26 | 56786 | 40865 | 30-Jan-2020 |  | J-16S-02-26 | 77228 | 55429 | 29-Jan-2020 |
| SP-16S-02-27 | 48862 | 34273 | 8-Feb-2020 |  | J-16S-02-27 | 34627 | 22316 | 6-Feb-2020 |
| SP-16S-02-28 | 55522 | 38426 | 12-Feb-2020 |  | J-16S-02-28 | 69170 | 45908 | 12-Feb-2020 |
| SP-16S-02-29 | 63624 | 44311 | 19-Feb-2020 |  | J-16S-02-29 | 62616 | 44600 | 17-Feb-2020 |
| SP-16S-02-30 | 13149 | 8849 | 28-Feb-2020 |  | J-16S-02-30 | N/A | N/A | 26-Feb-2020 |
| SP-16S-02-31 | 13912 | 9686 | 5-Mar-2020 |  | J-16S-02-31 | 11343 | 7389 | 4-Mar-2020 |
| SP-16S-02-32 | 11405 | 7366 | 12-Mar-2020 |  | J-16S-02-32 | 8199 | 5198 | 11-Mar-2020 |
| SP-16S-02-33 | 12227 | 7772 | 21-Mar-2020 |  | J-16S-02-33 | 13612 | 8928 | 19-Mar-2020 |
| SP-16S-02-34 | 13977 | 8840 | 12-Jul-2020 |  | J-16S-02-34 | 24234 | 17731 | 13-Jul-2020 |
| SP-16S-02-35 | 17892 | 10326 | 16-Jul-2020 |  | J-16S-02-35 | 10249 | 6212 | 15-Jul-2020 |
| SP-16S-02-36 | 74075 | 51573 | 31-Jul-2020 |  | J-16S-02-36 | 62155 | 45024 | 29-Jul-2020 |
| SP-16S-02-37 | 88759 | 56868 | 19-Aug-2020 |  | J-16S-02-37 | 58903 | 41490 | 20-Aug-2020 |
| SP-16S-02-38 | 57350 | 39239 | 23-Aug-2020 |  | J-16S-02-38 | 48567 | 34225 | 21-Aug-2020 |
| SP-16S-02-39 | 78415 | 52859 | 5-Sep-2020 |  | J-16S-02-39 | 81528 | 58179 | 3-Sep-2020 |
| SP-16S-02-40 | 84705 | 58691 | 22-Sep-2020 |  | J-16S-02-40 | 90321 | 62836 | 22-Sep-2020 |
| SP-16S-02-41 | 64870 | 47634 | 25-Sep-2020 |  | J-16S-02-41 | 45837 | 32093 | 25-Sep-2020 |
| SP-16S-02-42 | 28517 | 20417 | 11-Oct-2020 |  | J-16S-02-42 | 27660 | 19890 | 9-Oct-2020 |
| SP-16S-02-43 | 70195 | 47997 | 23-Oct-2020 |  | J-16S-02-43 | 73645 | 52318 | 22-Oct-2020 |
| SP-16S-02-44 | 77823 | 53157 | 10-Nov-2020 |  | J-16S-02-44 | 88173 | 64562 | 7-Nov-2020 |
| SP-16S-02-45 | 33170 | 22338 | 4-Dec-2020 |  | J-16S-02-45 | 81779 | 55574 | 3-Dec-2020 |
| SP-16S-02-46 | 29550 | 21395 | 20-Dec-2020 |  | J-16S-02-46 | 28378 | 20577 | 18-Dec-2020 |
| SP-16S-02-47 | 44947 | 28121 | 8-Jan-2021 |  | J-16S-02-47 | 24348 | 16499 | 7-Jan-2021 |
| SP-16S-02-48 | 25975 | 17809 | 14-Jan-2021 |  | J-16S-02-48 | 28903 | 20620 | 13-Jan-2021 |
| SP-16S-02-49 | 24802 | 16422 | 22-Jan-2021 |  | J-16S-02-49 | 31302 | 22012 | 20-Jan-2021 |
| SP-16S-02-50 | 20698 | 14503 | 29-Jan-2021 |  | J-16S-02-50 | 25432 | 18486 | 28-Jan-2021 |
| SP-16S-02-51 | 25927 | 14823 | 3-Feb-2021 |  | J-16S-02-51 | 24718 | 19006 | 5-Feb-2021 |
| SP-16S-02-52 | 19198 | 13574 | 12-Feb-2021 |  | J-16S-02-52 | 28336 | 21087 | 10-Feb-2021 |
| SP-16S-02-53 | 22555 | 12850 | 19-Feb-2021 |  | J-16S-02-53 | 23384 | 15098 | 20-Feb-2021 |
| SP-16S-02-54 | 23133 | 12876 | 23-Feb-2021 |  | J-16S-02-54 | 25181 | 16545 | 22-Feb-2021 |
| SP-16S-02-55 | 9158 | 2682 | 3-Mar-2021 |  | J-16S-02-55 | 11960 | 3777 | 3-Mar-2021 |
| SP-16S-02-56 | 10874 | 3222 | 13-Mar-2021 |  | J-16S-02-56 | 15106 | 5766 | 13-Mar-2021 |

**S3 Table. Sequence summary – 16S rRNA metabarcoding analysis (particle-associated bacteria) performed on Isla Julia (right) and Isla San Pedro (left) waters.**

| **ID** | **input** | **final** | **samples** |  | **Sample Name** | **input** | **final** | **samples** |
| --- | --- | --- | --- | --- | --- | --- | --- | --- |
| SP-16S-1-01 | 80410 | 53819 | 21-Mar-2019 |  | J-16S-1-01 | 103,548 | 80,782 | 20-Mar-2019 |
| SP-16S-1-02 | 67538 | 42191 | 2-Apr-2019 |  | J-16S-1-02 | 98,459 | 70,972 | 31-Mar-2019 |
| SP-16S-1-03 | 78685 | 50504 | 11-Apr-2019 |  | J-16S-1-03 | 111,746 | 79,681 | 11-Apr-2019 |
| SP-16S-1-04 | 87131 | 51175 | 16-Apr-2019 |  | J-16S-1-04 | 97,130 | 67,273 | 17-Apr-2019 |
| SP-16S-1-05 | 96201 | 66290 | 1-May-2019 |  | J-16S-1-05 | 92,432 | 65,778 | 28-Apr-2019 |
| SP-16S-1-06 | 176734 | 101410 | 7-May-2019 |  | J-16S-1-06 | 161,294 | 110,895 | 8-May-2019 |
| SP-16S-1-07 | 102109 | 58386 | 23-May-2019 |  | J-16S-1-07 | 108,189 | 81,167 | 25-May-2019 |
| SP-16S-1-08 | 142776 | 98124 | 2-Jun-2019 |  | J-16S-1-08 | 117,206 | 91,053 | 30-May-2019 |
| SP-16S-1-09 | 118366 | 80864 | 13-Jun-2019 |  | J-16S-1-09 | 49,021 | 35,172 | 14-Jun-2019 |
| SP-16S-1-10 | 141164 | 93115 | 19-Jun-2019 |  | J-16S-1-10 | 81,553 | 58,983 | 27-Jun-2019 |
| SP-16S-1-11 | 59604 | 42485 | 1-Jul-2019 |  | J-16S-1-11 | 100,627 | 77,877 | 29-Jun-2019 |
| SP-16S-1-12 | 75961 | 52596 | 6-Aug-2019 |  | J-16S-1-12 | 48,764 | 36,519 | 8-Aug-2019 |
| SP-16S-1-13 | 109209 | 39748 | 23-Aug-2019 |  | J-16S-1-13 | 33,435 | 16,050 | 21-Aug-2019 |
| SP-16S-1-14 | 151035 | 70213 | 11-Sep-2019 |  | J-16S-1-14 | 21,800 | 12,854 | 11-Sep-2019 |
| SP-16S-1-15 | 101667 | 43669 | 27-Sep-2019 |  | J-16S-1-15 | 23,525 | 11,821 | 25-Sep-2019 |
| SP-16S-1-16 | 69612 | 33579 | 7-Oct-2019 |  | J-16S-1-16 | 31,104 | 16,005 | 9-Oct-2019 |
| SP-16S-1-17 | 51972 | 35663 | 17-Oct-2019 |  | J-16S-1-17 | 15,071 | 10,943 | 18-Oct-2019 |
| SP-16S-1-18 | 57083 | 37865 | 15-Nov-2019 |  | J-16S-1-18 | 160,208 | 123,733 | 13-Nov-2019 |
| SP-16S-1-19 | 57245 | 37610 | 23-Nov-2019 |  | J-16S-1-19 | 111,477 | 88,735 | 23-Nov-2019 |
| SP-16S-1-20 | 63864 | 44812 | 1-Dec-2019 |  | J-16S-1-20 | 108,479 | 82,850 | 1-Dec-2019 |
| SP-16S-1-21 | 43896 | 27587 | 10-Dec-2019 |  | J-16S-1-21 | 110,402 | 83,685 | 8-Dec-2019 |
| SP-16S-1-22 | 29741 | 19647 | 17-Dec-2019 |  | J-16S-1-22 | 24,703 | 17,894 | 16-Dec-2019 |
| SP-16S-1-23 | 46473 | 32788 | 4-Jan-2020 |  | J-16S-1-23 | 14,657 | 10,199 | 3-Jan-2020 |
| SP-16S-1-24 | 20023 | 13540 | 14-Jan-2020 |  | J-16S-1-24 | 20,204 | 14,472 | 12-Jan-2020 |
| SP-16S-1-25 | 28733 | 21657 | 24-Jan-2020 |  | J-16S-1-25 | 14,558 | 10,624 | 23-Jan-2020 |
| SP-16S-1-26 | 80134 | 58021 | 30-Jan-2020 |  | J-16S-1-26 | 201,701 | 148,745 | 29-Jan-2020 |
| SP-16S-1-27 | 45763 | 34930 | 8-Feb-2020 |  | J-16S-1-27 | 17,172 | 10,793 | 6-Feb-2020 |
| SP-16S-1-28 | 47158 | 32356 | 12-Feb-2020 |  | J-16S-1-28 | 40,898 | 28,089 | 12-Feb-2020 |
| SP-16S-1-29 | 63603 | 47201 | 19-Feb-2020 |  | J-16S-1-29 | 85,116 | 61,794 | 17-Feb-2020 |
| SP-16S-1-30 | 10237 | 7375 | 28-Feb-2020 |  | J-16S-1-30 | 15,800 | 11,433 | 26-Feb-2020 |
| SP-16S-1-31 | 21100 | 13582 | 5-Mar-2020 |  | J-16S-1-31 | 16,906 | 13,238 | 4-Mar-2020 |
| SP-16S-1-32 | 14630 | 10078 | 12-Mar-2020 |  | J-16S-1-32 | 22,642 | 17,571 | 11-Mar-2020 |
| SP-16S-1-33 | 18335 | 11099 | 21-Mar-2020 |  | J-16S-1-33 | 29,644 | 23,217 | 19-Mar-2020 |
| SP-16S-1-34 | 13336 | 9261 | 12-Jul-2020 |  | J-16S-1-34 | 20,271 | 15,195 | 13-Jul-2020 |
| SP-16S-1-35 | 20193 | 13629 | 16-Jul-2020 |  | J-16S-1-35 | 31,505 | 21,603 | 15-Jul-2020 |
| SP-16S-1-36 | 52118 | 36604 | 31-Jul-2020 |  | J-16S-1-36 | 113,137 | 80,295 | 29-Jul-2020 |
| SP-16S-1-37 | 21500 | 16011 | 19-Aug-2020 |  | J-16S-1-37 | 37,555 | 29,528 | 20-Aug-2020 |
| SP-16S-1-38 | 49440 | 36371 | 23-Aug-2020 |  | J-16S-1-38 | 85,949 | 50,520 | 21-Aug-2020 |
| SP-16S-1-39 | 38014 | 26695 | 5-Sep-2020 |  | J-16S-1-39 | 51,680 | 41,712 | 3-Sep-2020 |
| SP-16S-1-40 | 58155 | 43674 | 22-Sep-2020 |  | J-16S-1-40 | 40,885 | 29,971 | 22-Sep-2020 |
| SP-16S-1-41 | 53042 | 37228 | 25-Sep-2020 |  | J-16S-1-41 | 39,844 | 30,671 | 25-Sep-2020 |
| SP-16S-1-42 | 21274 | 16562 | 11-Oct-2020 |  | J-16S-1-42 | 26,667 | 21,171 | 9-Oct-2020 |
| SP-16S-1-43 | 60788 | 44732 | 23-Oct-2020 |  | J-16S-1-43 | 65,462 | 50,569 | 22-Oct-2020 |
| SP-16S-1-44 | 49889 | 36799 | 10-Nov-2020 |  | J-16S-1-44 | 63,301 | 47,979 | 7-Nov-2020 |
| SP-16S-1-45 | 56980 | 39233 | 4-Dec-2020 |  | J-16S-1-45 | 64,725 | 49,105 | 3-Dec-2020 |
| SP-16S-1-46 | 19653 | 13050 | 20-Dec-2020 |  | J-16S-1-46 | 33,081 | 26,289 | 18-Dec-2020 |
| SP-16S-1-47 | 23474 | 16437 | 8-Jan-2021 |  | J-16S-1-47 | 46,214 | 39,375 | 7-Jan-2021 |
| SP-16S-1-48 | 23265 | 17870 | 14-Jan-2021 |  | J-16S-1-48 | 34,367 | 25,704 | 13-Jan-2021 |
| SP-16S-1-49 | 24566 | 17965 | 22-Jan-2021 |  | J-16S-1-49 | 31,360 | 22,373 | 20-Jan-2021 |
| SP-16S-1-50 | 32448 | 22278 | 29-Jan-2021 |  | J-16S-1-50 | 35,278 | 26,973 | 28-Jan-2021 |
| SP-16S-1-51 | 32563 | 20367 | 3-Feb-2021 |  | J-16S-1-51 | 24,070 | 17,857 | 5-Feb-2021 |
| SP-16S-1-52 | 27662 | 18945 | 12-Feb-2021 |  | J-16S-1-52 | 25,408 | 22,205 | 10-Feb-2021 |
| SP-16S-1-53 | 20415 | 15561 | 19-Feb-2021 |  | J-16S-1-53 | 28,186 | 17,935 | 20-Feb-2021 |
| SP-16S-1-54 | 23151 | 15279 | 23-Feb-2021 |  | J-16S-1-54 | 23,799 | 18,071 | 22-Feb-2021 |
| SP-16S-1-55 | 7999 | 3222 | 3-Mar-2021 |  | J-16S-1-55 | 15,059 | 8,838 | 3-Mar-2021 |
| SP-16S-1-56 | 11396 | 7136 | 13-Mar-2021 |  | J-16S-1-56 | 16,501 | 8,221 | 13-Mar-2021 |

**S4 Table. Bacterial genus composition in *A. catenella* culture before and after antibiotic treatment analyzed by 16S rRNA gene metabarcoding assay (decreased relative % after treatment represent by red).**

| **Genus** | **Pre, %** | **Post, %** | **>5% increase** |
| --- | --- | --- | --- |
| Amilibacter | 0.00 | 0.13 |  |
| Antarctobacter | 0.00 | 0.41 |  |
| Aurantivirga | 0.00 | 0.03 |  |
| Aliiglaciecola | 17.60 | 0.00 |  |
| Alteromonas | 1.19 | 0.00 |  |
| Antarctobacter | 0.51 | 0.00 |  |
| Aquimixticola | 0.42 | 0.00 |  |
| Balneola | 0.57 | 0.64 |  |
| Bdellovibrio | 1.35 | 0.00 |  |
| Bermanella | 0.83 | 0.00 |  |
| Catenococcus | 0.00 | 0.01 |  |
| Cephaloticoccus | 0.05 | 0.00 |  |
| Coxiella | 0.00 | 0.73 |  |
| Fabibacter | 0.16 | 0.45 |  |
| Haliea | 0.20 | 0.00 |  |
| Hoeflea | 0.33 | 0.00 |  |
| Kordiimonas | 0.00 | 0.56 |  |
| Leisingera | 0.99 | 0.00 |  |
| Maricaulis | 9.57 | 0.00 |  |
| Marinobacter | 18.95 | 0.87 |  |
| Marinoscillum | 0.75 | 1.80 |  |
| Marivita | 1.07 | 0.00 |  |
| Methylophaga | 3.20 | 0.43 |  |
| Methylotenera | 0.05 | 0.00 |  |
| Mf105b01 | 0.18 | 0.20 |  |
| NA | 28.25 | 15.53 |  |
| Nisaea | 0.70 | 0.18 |  |
| Oleiphilus | 0.13 | 0.00 |  |
| **Paraglaciecola** | **0.00** | **49.87** | **Yes** |
| Pir4_lineage | 0.00 | 0.04 |  |
| Polycyclovorans | 0.19 | 0.09 |  |
| Porticoccus | 0.30 | 0.00 |  |
| Pseudohongiella | 0.58 | 0.46 |  |
| **Reichenbachiella** | **0.20** | **10.01** | **Yes** |
| Rhodococcus | 0.00 | 0.10 |  |
| Rhodopirellula | 0.05 | 0.00 |  |
| Saccharospirillum | 0.44 | 0.45 |  |
| SM1A02 | 9.39 | 0.00 |  |
| Sphingorhabdus | 0.08 | 0.37 |  |
| **Spongiibacter** | **0.67** | **10.74** | **Yes** |
| Tenacibaculum | 0.26 | 0.00 |  |
| **Thalassospira** | **0.79** | **5.89** | **Yes** |

**S1 Fig. Bacterial composition in *Alexandrium catenella* culture before and after antibiotic treatment analyzed by 16S rRNA gene metabarcoding assay.**


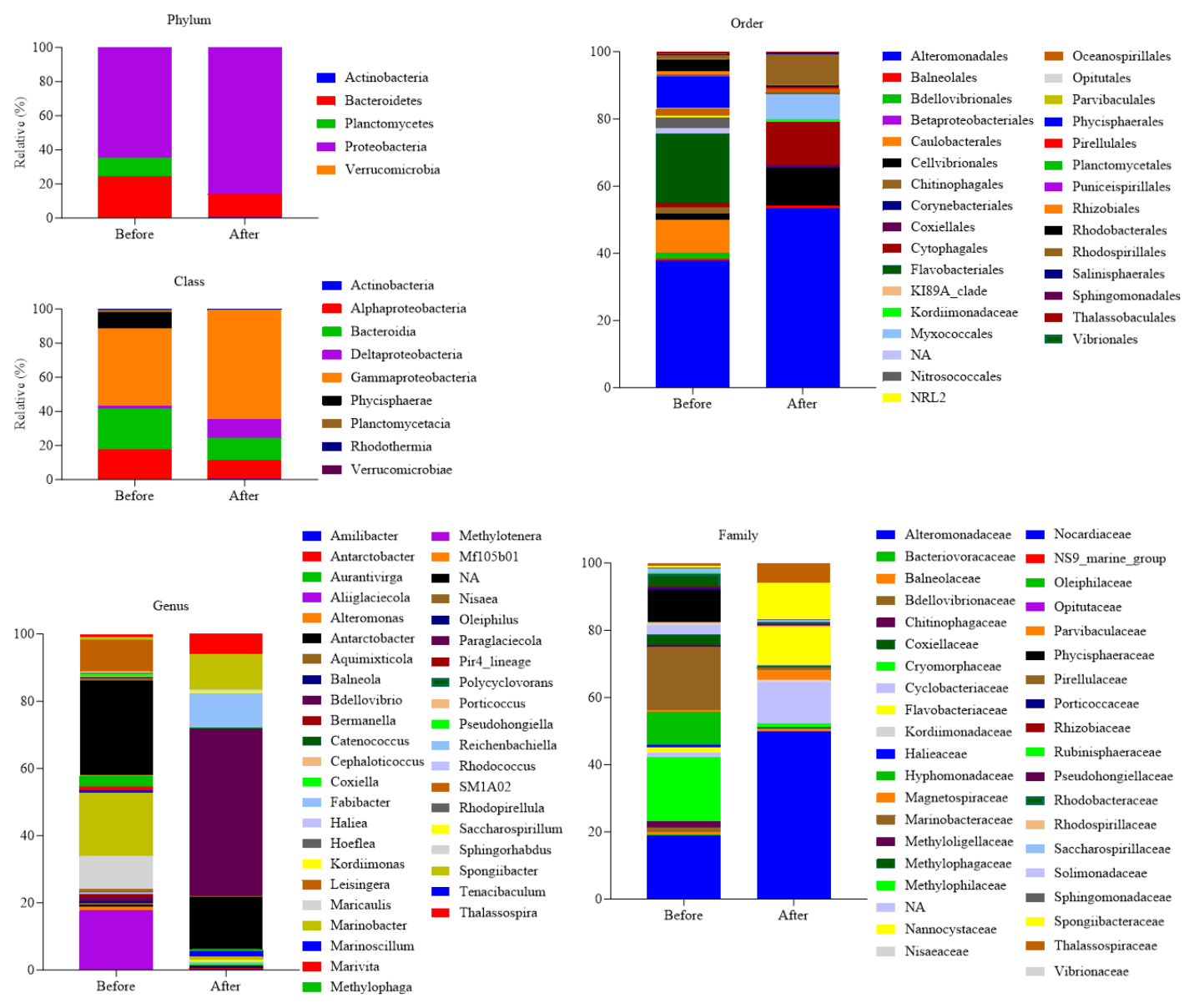


**S2 Fig. Microscopic analysis of phytoplankton species in Isla Julia on 10/22/2022 and Isla San Pedro on 10/23/2022 (total cell counts per ml).**

**S3 Fig. Time lag relationship between Spongiibacteracea and Alexandrium spp. in Melinka by Cross-correlations.**

Melinka: Free-living Spongiibacteracea and Alexandrium


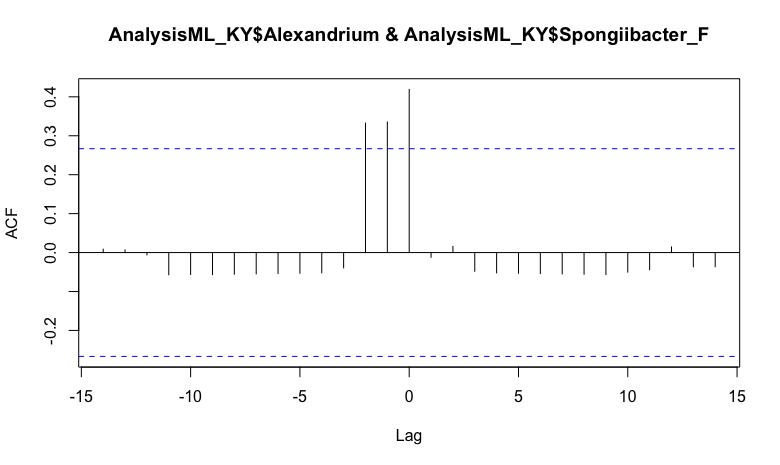


Melinka: Attached Spongiibacteracea and Alexandrium


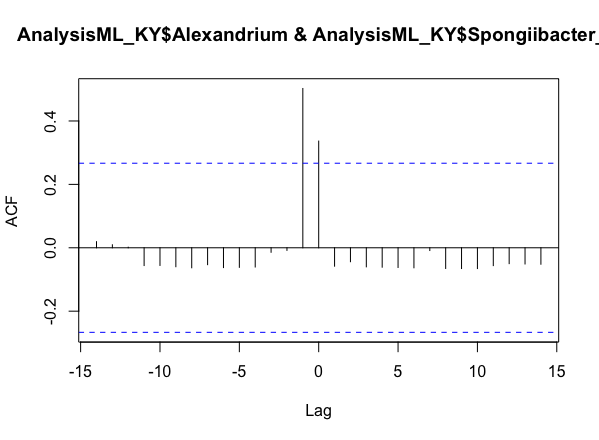

Supplement: S1 File — (DOCX) [file pone.0301343.s001.docx]
